# Hyperbaric oxygenation of autoimmune manifestation in coversational disorders

**DOI:** 10.1192/j.eurpsy.2021.404

**Published:** 2021-08-13

**Authors:** D. Labunskiy, S. Kiryukhina, V. Podsevatkin, E. Govsh, V. Kolmyykov

**Affiliations:** Neurology And Psychiatry, Ogarev Mordovia State University, Saransk, Russian Federation

**Keywords:** Conversional Disorders, Hyperbaric Oxygenaations, Immunoglobulins

## Abstract

**Introduction:**

Study of conversion disorders is urgent problem in psychiatry due to high prevalence of hysterical manifestations, both in structure of various mental diseases and in general somatic network: among population it is from 0.5 to 2%.

**Objectives:**

Our aim was to study the effect of complex therapy, combining traditional psychopharmacological drugs and hyperbaric oxygenation, on indicators of acid-base balance of blood, neurotransmitter metabolism, immune and hormonal status in experimental modeling of stress, as well as reduction of psychopathological symptoms in various forms of hysterical disorders.

**Methods:**

Studies were conducted with the participation of 160 patients (145 women and 15 men), average age 33.5 ± 6.1 years, Сontent of adrenaline, norepinephrine, dopamine, serotonin was determined by concentration of prolactin, thyroid-stimulating hormone (TSH), free thyroxine (T4 light), cortisol using ELISA. Immune status was assessed according to following indicators: determination of level of immunoglobulins of classes A, M and G by the method of radial immunodiffusion in a gel; study of total complementary activity of blood serum by hemolytic method.

**Results:**

It is necessary to highlight a significant increase in the concentration of Ig G and Ig A, a higher level of large, medium and small circulating immune complexes, which does not exclude the development of autoimmune reactions as a result of a long course of the mental process, which occurs with damage to the own cells of the nervous tissue.

**Conclusions:**

Кevealed changes in the immune and endocrine reactions upon admission, under the influence of HBO treatment indicate involvement of these structures in the pathogenetic mechanisms.

**Disclosure:**

No significant relationships.

